# Prostate Volume Influence on Postoperative Outcomes for Patients Undergoing RARP: A Monocentric Serial Analysis of 500 Cases

**DOI:** 10.3390/jcm12072491

**Published:** 2023-03-25

**Authors:** Mahmoud Farzat, Josef Rosenbauer, Christian Tanislav, Florian M. Wagenlehner

**Affiliations:** 1 Department of Urology and Robotic Urology, Diakonie Klinikum Siegen, Academic Teaching Hospital of the University of Bonn, 53127 Bonn, Germany; 2 Department of Urology, Pediatric Urology and Andrology, Justus-Liebig University of Giessen, 35390 Giessen, Germany; 3 Department of Geriatric and Neurology, Diakonie Klinkum Siegen, Academic Teaching Hospital of the University of Bonn, 53127 Bonn, Germany

**Keywords:** prostate cancer, RARP, prostate volume

## Abstract

Elevated prostate volume is considered to negatively influence postoperative outcomes after robot-assisted radical prostatectomy (RARP). We aim to investigate the influence of prostate volume on readmissions and complications after RARP. Methods: A total of 500 consecutive patients who underwent RARP between April 2019 and August 2022 were included. Patients were dichotomized into two groups using a prostate volume cut-off of 50 mL (small and normal prostate (SNP) n = 314, 62.8%; large prostate n = 186, 37.2%). Demographic, baseline, and perioperative data were analyzed. The postoperative complications and readmission rates within 90 days after RARP were compared between groups. A univariate linear analysis was performed to investigate the association between prostate volume and other relevant outcomes. Results: Patients with larger prostates had a higher IPSS score, and therefore, more relevant LUTS at the baseline. They had higher ASA scores (*p* = 0.015). They also had more catheter days (mean 6.6 days for SNP vs. 7.5 days for LP) (*p* = 0.041). All oncological outcomes were similar between the groups. Although statistical analysis showed no significant difference between the groups (*p* = 0.062), a trend for minor complications in patients with larger prostates, n = 37/186 (19.8%) for the LP group vs. n = 37/314 (11.7%) in the SNP group, was observed. Namely, acute urinary retention and secondary anastomosis insufficiency. Major complications with an SNP (4.4%) and LP (3.7%) (*p* = 0.708) and readmissions with an SNP (6.25%) and LP (4.2%) (*p* = 0.814) were infrequent and distributed equally between the groups. In univariate analysis, prostate volume could solely predict a longer console time (*p* = 0.005). Conclusions: A higher prostate volume appears to have minimal influence on the perioperative course after RARP. It can prolong catheter days and increase the incidence of minor complications such as acute urinary retention. However, it might predict minor changes in operating time. Yet, prostate volume has less influence on major complications, readmissions, or oncological results.

## 1. Introduction

Robot-assisted radical prostatectomy and radiotherapy are the standard local therapies used to actively treat prostate carcinoma [1]. While the use of radiotherapy in small prostates is rarely contraindicated, its application in larger prostates may necessitate a different approach, including surgical deobstruction in parallel [1]. On the contrary, prostate size is not a contraindication for robot-assisted radical prostatectomy (RARP) [2]. In general, an elevated prostate volume, among other baseline parameters such as obesity, higher PSA, prostate pretreatment, and comorbidities, is considered to negatively influence the postoperative outcomes after prostatectomy [3]. The specific influence of prostate volume on the prostatectomy outcomes is widely studied [4]. Regarding patient-reported outcome measures (PROMs), the operative technique, rather than prostate volume, influenced the results in a robotic simple prostatectomy population [5]. Conversely, lower urinary tract symptoms (LUTS), age, the extent of nerve-sparing surgery, and surgeon experience influenced PROMS such as post-prostatectomy incontinence in the RARP cohort [6]. Various publications describe how large prostates increase the console time, cause more blood loss, and lead to a higher rate of complications [7,8]. Other urologists reported favorable oncological outcomes for larger prostates after open, laparoscopic, and robot-assisted prostatectomy [2,7,9]. However, the impact of prostate volume in a monocentric, single-surgeon cohort of 500 patients, 40% of whom had a locally advanced tumor, on readmissions and postoperative complications has not been thoroughly investigated. Our current study aims to explore the influence of preoperatively estimated prostate volume on transrectal ultrasound (TRUS) from a clinical point of view in a large in-hospital database.

## 2. Materials and Methods

### 2.1. Surgical Procedure and Setting

All procedures (n = 500) were completed using a transperitoneal approach with the Da Vinci X^®^ Surgical System (Intuitive Surgical, Sunnyvale, CA, USA). Pelvic lymphadenectomy was performed in all patients, and no intraabdominal drainage was inserted. Prior to skin incision, intravenous single-shot antibiotics were administered. The vesicourethral anastomosis (VUA) was performed in a one-layer fashion with a continuous circumferential double-armed barbed suture. In most cases, the anastomosis included a one-layer Rocco stitch. After completion, the patients intraoperatively received an anastomosis water-tightness test with 200–300 mL of sterile NaCl. All patients received a transurethral catheter (TUC) and a suprapubic catheter (SPC). The transurethral catheter was removed on the first postoperative day (POD1). On POD3, the patients were allowed to urinate naturally. The suprapubic catheter was removed one day after successful micturition without post-void residual urine. In cases of primary extravasation on cystography, patients were discharged with the catheters, which were removed in an outpatient visit a couple of days later.

### 2.2. Participants and Methods

A total of 500 consecutive patients from a prospectively acquired database who underwent RARP between April 2019 and August 2022 by a specialized surgeon due to locally confined (pT2; n = 295; 59.4%) and locally advanced prostate cancer (pT3–4; n = 203; 40.6%) were included in this analysis. Patients were dichotomized into two groups based on their prostate volume using a cutoff value of 50 mL measured by preoperative transrectal ultrasound (TRUS): the small and normal prostate (SNP) group had values under or equal to 50 mL, and the large prostate group has values above 50 mL, respectively. Demographic, intraoperative, and postoperative data were analyzed and compared using propensity score matching. The variables included were age, international prostate symptom score (IPSS), international index of sexual function (IIEF), initial PSA, pre- and postoperative Gleason scores, prostate volume, body mass index (BMI), the American Association of Anesthesiology Morbidity Score (ASA), pre- and postoperative hemoglobin differences (Hgb), previous medical and surgical treatment of the prostate, and D’Amico Risk Classification. Postoperative complications within 90 days after RARP were graded by the Clavien–Dindo classification [10]. Readmission rate was considered to be the primary endpoint, and complications were the secondary endpoint. A univariate linear regression analysis to investigate the association between prostate volume and readmissions, among other perioperative outcomes, was also carried out.

Statistical analysis was performed using SPSS^®^ v27. Categorical variables were summarized as frequencies (percentage) and continuous variables as mean ± standard deviation, interquartile ranges (IQR), and median values. The Kolmogorov–Smirnov one-sample test was used to verify normal distribution. Matched pair analysis using the independent T-test for parametric numeric variables and the Mann–Whitney U-test for nonparametric variables was performed. A Pearson Chi Square test was also used to compare the relative frequencies. Univariable logistic regression and linear regression models were used for further association analysis.

### 2.3. Ethics Statement

The study was conducted in accordance with the ethical standards of the Declaration of Helsinki and approved by the ethics committee of the medical association Westfalen-Lippe and Wilhelm’s University of Münster (2022-585-f-S).

## 3. Results

### 3.1. Baseline Parameters

The median prostate volume in the SNP group was 35 mL, whereas it was 67 mL in the LP group. Initial PSA values were higher in the LP group (16.7 vs. 13.5 ng/mL; *p* = 0.004). Patients with larger prostates suffered more from LUTS (IPSS scores of 9.7 vs. 14.7 for SNP and LP, respectively; *p* < 0.001), and they had more comorbidities since their ASA score was higher (*p* = 0.015). BMIs were different between the groups, with a median of 28 in LP patients vs. a median of 27 in the SNP group (*p* = 0.049). Almost two-thirds of the men in our series were operated on using the nerve sparing technique, with the majority being bilaterally spared. Unilateral or partial nerve sparing were less frequent among the two study groups. All other baseline clinical and oncological parameters were comparable between the groups (Table 1).

### 3.2. Intraoperative Data

The median prostate weight in the final pathology report was 49 g for the SNP group and 76 g for the LP group. This included the weight of both seminal vesicles. The difference in mean console time was only 2 min between the groups, in favor of the SNP group, without any significant difference (*p* = 0.653), while the median console operating time was 140 min for both groups. The mean number of suprapubic catheter days in the LP group was 7.5 days versus 6.67 days in the SNP group, despite there being a median of 5 days in both cohorts. Statistical analysis revealed a significant difference (*p* = 0.041). The hemoglobin difference between preoperative and postoperative hemoglobin values was higher in men with larger prostates (2.7 g/dL in the LP group vs. 2.5 g/dL in the SNP group; *p* = 0.004). Tumor stages, Gleason score distributions, as well as positive surgical margins, were similar between the groups. The details are in Table 2.

### 3.3. Complications and Readmissions

Despite a trend for minor complications in the patients larger prostates (n = 37/186 (19.8%) for the LP group vs. n = 37/314 (11.7%) in the SNP group), the statistical analysis showed no significant difference between groups (*p* = 0.062). The most frequent minor complication (e.g., Clavien–Dindo I) to be reported was acute urinary retention, with an overall incidence of n = 28/500 (5.6%) and a noteworthy difference between the groups with LPs (n = 15/186 (8%) vs. 13/314 (4.1%) in the SNP group). The most recurrent Clavien–Dindo II complications to be monitored were secondary vesicourethral anastomosis leakage (SVUAL) and urinary tract infections (UTIs). While UTIs were equally distributed with similar incidences in both groups (2.2%), the SVUAL values were higher in LP patients (n = 9/184 (4.8%) vs. n = 2/314 (0.6%)). Major complications and readmissions were similarly distributed between the groups. Grad IIIa complications characterize interventions carried out under local anesthesia, mostly symptomatic lymphocele (overall n = 10/500; 2%). In those cases, a drain was inserted percutaneously and removed after a couple of days. One patient with a long history of coronary heart disease and an uneventful intra- and postoperative course presented one week after discharge with an NSTEMI and received coronary stents without further complications. Three men experienced an upper urinary tract obstruction (UTTO; n = 3, 0.6%), in which a temporary DJ catheter was inserted under general anesthesia. A total of five patients had to be re-operated on for bleeding (n = 1), ileus (n = 1), and incisional hernias (n = 2). Another obese patient with a history of low anterior resection of the rectum received a stoma due to necrosis of the bowel pouch 1 month after the initial RARP. One CD IV complication was recorded in an obese patient with a T4 tumor who developed Rhabdomyolysis without compartment syndrome. The patient was admitted to our intensive care unit and received hemodialysis. The Rhabdomyolysis was resolved completely without leaving permanent lesions. After discharge, (n = 28) 5.6% of the patients were readmitted within 90 days after RARP. No differences were observed between the groups (*p* = 0.814). Further details are in Table 3.

A univariate linear and logistic regression analysis revealed a relationship between prostate volume and prolonged console time (*p* = 0.005). However, prostate volume could not independently predict elevated readmission or complication rates, nor catheter days or hospital stays. Furthermore, no correlation was found with positive surgical margins, transfusions, or lymphoceles. The details are given in Table 4.

## 4. Discussion

The major finding in our research is that an increased prostate volume in experienced centers does not have a negative impact on clinically important outcomes such as major complications and readmissions. However, it may prolong the operative time (only 2 min in our cohort means 150 vs. 152 min) and the number of catheter days. Moreover, it increases intraoperative blood loss without having clinical implications. Furthermore, from an oncological point of view, patients with larger prostates did not have poorer results. We also found that the pre-estimated prostate volume in TRUS can independently predict a longer console time (*p* = 0.005).

In general, an elevated prostate volume, among other baseline parameters such as obesity, higher PSA, prostate pretreatment, and comorbidities, is considered to negatively influence the postoperative outcomes after prostatectomy [3]. Specifically, prostatectomy in men with large prostates is thought to be accompanied by more blood loss, as well as complications, yet favorable oncological results [4]. In total, (n = 28/500) 5.6% of the men in our cohort were readmitted within 90 days after RARP. Our rates are in line with those in other reports [11,12]. Only n = 7/186 (4.2%) patients were readmitted in the LP group versus n = 21/314 (6.25%) in the SNP group. Statistically, no difference was noted (*p* = 0.814).

While Hirasawa et al. reported increased blood loss and perioperative complications with increasing prostate volume [8]. In our study, complications were equally distributed among the groups, despite a trend for minor complications in men with larger prostates (*p* = 0.062), namely acute urinary retention (AUR), in which the existing suprapubic catheter remained in place for a longer period of time and no further measures were needed. The other prominent minor complication was secondary vesicourethral anastomosis leakage (VAUL), n = 9/186 (4.8%). This is in line with other reports [8,9,11].

Other authors reported more favorable oncological outcomes in their patients compared to those of patients with smaller prostates [2,8,13]. Residual benign prostate glandular tissue in men undergoing radical prostatectomy is found in 23–29% of cases [14,15]. It was more frequent in younger men undergoing the robot-assisted nerve sparing technique [14]. Greenberg et al. found that benign glandular margins (BGM) were not associated with an increased risk of malignancy at the surgical margin MSM, detectable PSA, biochemical recurrence (BCR), or progression after detectable PSA [14].

Although 40.6% of our cohort had locally advanced tumors, we found positive surgical margins (total n = 36/500, 7.2%), Gleason score distributions, and the tumor stages to be similar among our study groups. Despite the fact that almost two-thirds of the men in our cohort underwent the nerve sparing technique, we renounced the use of frozen sections since non-focal positive surgical margins (>3 mm) [16] were uncommon and were found only in 3.2% and 4.3% of the SNP and LP groups, respectively. Nevertheless, the use of fluorescence confocal microscopy in prostate cancer diagnostics [17], as well as in the form of digital frozen sections, is found to be very promising [18] and can be considered in the future to improve the oncological outcomes.

The presence of variant histologies in prostate cancer patients may implicate worse biochemical recurrence and cancer-specific mortality [19]. We reported our results considering the most common and predominant type of prostate cancer found in our cohort, namely adenocarcinoma. The most common variant histology reported by our pathologist was mucinous prostate carcinoma in n = 8/500, 1.6%, followed by ductal carcinoma in n = 6/500, 1.2%, and the neuroendocrine subtype in n = 4/500, 0.8%. In all these cases, it was admixed with invasive adenocarcinoma [20]. The presence of those rarer histological variants of prostate cancer implicated more strict postoperative follow-up regimes [21]. We did not encounter rarer histologies such as signet ring cell or adenosquamous in our patients.

In contrast to what Kim et al. found in their extraperitoneal cohort, we found that patients with a larger prostate size had a longer console time and more blood loss [7]. The mean console time was only 2 min shorter in men with smaller prostates (150 SNP vs. 152 min LP). This might also explain the low rate of complications such as thrombus and emboli in our cohort. Nonetheless, it implies a selection bias between the two study groups and a weakness of the serial comparison. This may also influence the oncological and functional results, as well as complications and readmissions after RARP. Therefore, we performed univariate analysis, which showed the prostate volume to correlate linearly with the console time.

Regarding intraoperative blood loss, we measured the difference between the preoperative and predischarge hemoglobin values. We found that patients with large prostates have a greater difference (2.7 g/dL vs. 2.5 g/dL; *p* = 0.004). Nevertheless, this did not involve a clinical consequence since only 1.2% (n = 7/500; *p* = 0.747) needed a transfusion. This is in accordance with the findings of other authors [7,8].

Interestingly, we noticed an almost 20% increase between the prostate volume on transrectal ultrasound and the prostate weight in the final pathological report (mean on TRUS 43 mL versus mean 55 g in the pathological report). Therefore, we ran Pearson’s correlation test between the two variables. It resulted in a positive correlation of 0.869 and a *p* value of <0.001, which represent a linear correlation, which can be partly explained through the inherent inaccuracy of the TRUS measurement, examiner-related bias, and the fact that the weight in the final pathological report included the weight of seminal vesicles, and sometimes, peri-prostatic tissues removed in cases of locally advanced tumors.

The strength of our investigation lies in the large number of patients included and the detailed analysis of their perioperative parameters and outcomes. Yet, the limitations must be taken into account. The main limitation of our study is the retrospective design. Secondly, we could not include long-term outcome measures due to the lack of follow-up data, which is partly explained by the practices of the German national health care system, in which a follow-up is not conducted by tertiary referral centers. The relevance of our study, and therefore its evidence base, must be narrowed since it was solely based on a densely occupied urological department and an expert surgeon. Moreover, among the patients undergoing RARP, differences in outcomes are associated with the patients’ complexity. Our collective includes a high proportion of locally advanced tumors (40.6%). Still, 96% of patients managed to be discharged uneventfully.

## 5. Conclusions

In experienced hands, a higher prostate volume has minimal influence on the postoperative course after RARP. However, it might predict minor changes in operating times and number of catheter days. Patients with prostate carcinoma should be offered RARP, regardless of their prostate size, since it can be delivered without increasing the risk of major adverse events or readmission rates. Still, operating on a larger prostate after finishing learning is advisable in order to avoid complications caused by longer OR times and greater blood loss.

## Figures and Tables

**Table 1:** Analysis of demographic, baseline clinical, and preoperative characteristics between groups.

	Total n = 500	Small and Normal Prostate under or Equal to 50 mL n = 314 (62.8%)	Large Prostate above 50 mL n = 186 (37.2%)	*p*-Value
Age (year)				0.479
Mean ± SD	66.8 ± 7.1	65.97 ± 7.3	68.25 ± 6.5	
IQR	62–72	61–71	63–73	
Median	68	67	70	
ASA-score				0.015
1	96 (19.2)	68 (21.7)	28 (15.1)	
2	314 (62.8)	198 (63.1)	116 (62.4)	
3	82 (16.4)	43 (13.7)	39 (21.0)	
Missing	8 (1.6)	5 (1.6)	3 (1.3)	
Preoperative HGB (g/dL)				0.358
Mean ± SD	14.7 ± 1.3	14.6 ± 1.36	14.7 ± 1.2	
IQR	14.1–15.5	14.0–15.5	14.1–15.6	
Median	14.8	14.8	14.9	
IPSS				<0.001
Mean ± SD	11.4 ± 8.3	9.7 ± 7.4	14.7 ± 8.9	
IQR	5–16	4–8	7–21	
Median	8.3	8	12	
IIEF				0.079
Mean ± SD	15.2 ± 8.7	15.8 ± 8.8	14.3 ± 8.4	
IQR	6–23	6–17	6–21.5	
Median	17	17	15	
Initial PSA (ng/mL)				0.004
Mean ± SD	14.8 ± 24.5	13.5 ± 19.6	16.7 ± 26.4	
IQR	5.5–13.6	5.3–12	5.9–16	
Median	8	7.5	9	
BMI				0.049
Mean ± SD	28.4 1 ± 4.3	28.1 ± 4.1	29 ± 4.9	
IQR	25–31	25–30	25.7–31	
Median	28	27	28	
Pretreatment				
Medical (hormonal therapy)	55 (11)	29	26	0.130
Surgical (TUR-P)	34 (6.8)	29	5	0.005
D’Amico Risk Classification				0.146
Low risk	117 (23.4%)	76 (24.2%)	41 (22%)	
Intermediate risk	229 (45.8%)	150 (47.8%)	79 (42.5%)	
High risk	154 (30.8%)	88 (28%)	66 (35.5%)	
Preoperative Gleason score				0.445
5	1 (0.2%)	1 (0.3%)	0	
6	140 (28%)	87 (27.7%)	53 (28.5%)	
3 + 4	176 (35.2%)	117 (37.3%)	59 (31.7%)	
4 + 3	59 (11.8%)	37 (11.8%)	22 (11.8%)	
8	82 (16.4%)	48 (15.3%)	34 (18.3%)	
9	36 (7.2%)	20 (6.4%)	16 (8.6%)	
10	5 (1.0%)	4 (1.3%)	1 (0.5%)	
Unclassified *	1 (0.2%)	0	1 (0.5%)	
Nerve Sparing				0.613
(bilateral)	347 (69.4%)	231 (73.6%)	116 (62.4%)	
(unilateral)	19 (3.8%)	6 (1.9%)	13 (7%)	
No	134 (26.8%)	77 (24.5%)	57 (30.6%)	

Categorical data are presented as numbers (%). SD: standard deviation; IQR: interquartile range; ASA: American Association of Anesthesiology comorbidity score; HGB: hemoglobin; BMI: Body mass index; IPSS: International Prostate Symptom Score; IIEF: International Index of Erectile Function; PSA: prostate-specific antigen; TUR-P: transurethral resection of the prostate. * Patients received androgen deprivation therapy before biopsy.

**Table 2:** Intra- and postoperative data and pathological findings for all groups.

	Total n = 500	Small and Normal Prostate under or Equal to 50 mL n = 314 (62.8%)	Large Prostateabove 50 mL n = 186 (37.2%)	*p*-Value
Console time (minute)				0.653
Mean ± SD	151 ± 45	150 ± 47	152 ± 42	
IQR	120–180	120–180	120–180	
Median	140	140	140	
Prostate weight (g)				<0.001
Mean ± SD	61 ± 25.6	49.3 ± 12.7	82.2 ± 28	
IQR	64–72	40–56.7	64–90	
Median	55	49	76	
Pathological stage				0.126
0	1 (0.2)	0	1 (0.5)	
pT1	1 (0.2)	1 (0.3)	0	
pT2	295 (59)	184 (58.6)	111 (59.7)	
pT3	183 (36.6)	121 (38.5)	62 (33.3)	
pT4	20 (4.0)	8 (2.5)	12 (6.5)	
Postoperative Gleason score				0.077
6	28 (5.6)	15 (4.8)	13 (7)	
3 + 4	282 (56.4)	189 (60.2)	93 (50)	
4 + 3	89 (17.8)	56 (17.8)	33 (17.7)	
8	26 (5.2)	11 (3.5)	15 (8.1)	
9	29 (5.8)	17 (5.4)	12 (6.5)	
10	1 (0.2)	1 (0.3)	0	
Unclassified *	45 (9.0)	25 (8)	20 (10.8)	
Positive surgical margins (total)	36 (7.2)	24 (7.6)	12 (6.5)	0.619
<3 mm	18 (3.6)	14 (4.5)	4 (2.2)	
>3 mm	18 (3.6)	10 (3.2)	8 (4.3)	
Number of Lymph nodes				0.413
Mean ± SD	19.6 ± 7.4	19.5 ± 7.4	20.1 ± 7.5	
IQR	(15–24)	(15–23)	(14–26)	
Median	18	18	19	
Positive Lymph node	87 (17.4%)	49 (15.6%)	38 (20.4%)	0.169
HGB Difference (g/dL)				0.004
Mean ± SD	2.5 ± 4.8	2.5 ± 4.8	2.7 ± 1.3	
IQR	1.9–3.5	(1.8–3.4)	(2–3.8)	
Median	2.6	2.6	2.6	
Transfusion	7 (1.2%)	4 (1.3%)	3 (1.5%)	0.747
Length of hospitalization (days)				0.490
Mean ± SD	5.6 ± 1.6	5.66 ± 1.26	5.55 ± 2	
IQR	(5–6)	(5–6)	(5–6)	
Median	5	5	5	
Catheter days				0.041
Mean ± SD	6.9 ± 4.7	6.67 ± 4.4	7.55 ± 5.2	
IQR	4–10	(4–8.5)	(4–10)	
Median	5	5	5	

Categorical data are presented as numbers (%). SD: standard deviation; IQR: interquartile range; HGB hemoglobin. * Patients received androgen deprivation therapy preoperatively.

**Table 3:** Thirty day complications and readmissions.

Complications in Detail			Total (n = 500)	Small and Normal Prostate under or Equal to 50 mL 314 (62.8%)	Large Prostate above 50 mL 186 (37.2%)	*p*-Value
	Minor		74 (14.8%)	37 (11.7%)	37 (19.8%)	0.062
Minor	CD I 51 (10.2)	Thrombus/Embolism	4 (0.8%)	3 (0.9%)	1 (0.5%)	
		Elevated Labor Parameter	6 (1.2%)	4 (1.2%)	2 (1%)	
		AUR	28 (5.6%)	13 (4.1%)	15 (8%)	
		Diverse	13 (2.6%)	7 (2.0%)	6 (3.6%)	
	CD II 23 (4.6)	Secondary VUAL *	11 (2.2%)	2 (0.6%)	9 (4.8%)	
		UTI	11 (2.2%)	7 (2.2%)	4 (2.2%)	
		Hematoma requiring Transfusion	1 (0.2%)	1 (0.3%)	0	
	Major		21 (4.2%)	14 (4.4%)	7 (3.7%)	0.708
Major	CD III a 12 (2.4)	Myocardial infarction	1 (0.2%)	1 (0.3%)	0	
		Hiatus Hernia	1 (0.2%)	1 (0.3%)	0	
		Symptomatic Lymphocele	10 (2.0%)	6 (1.9%)	4 (2.2%)	
	CD III b 8 (1.6)	Revision	5 (1.0%)	4 (1.2%)	1 (0.6%)	
		UUTO	3 (0.6%)	2 (0.6%)	1 (0.6%)	
	CD VI 1 (0.2)	Rhabdomyolysis	1 (0.2%)	0	1 (0.6%)	
		Readmissions *	28 (5.6%)	21 (6.25%)	7 (4.2%)	0.814

CD: Clavien–Dindo; AUR: acute urinary retention, VUAL: vesicourethral anastomosis leakage, UTI: urinary tract infection; UTTO: upper urinary tract obstruction. * Readmissions do not correlate linearly with complications, since some complications happened predischarge.

**Table 4:** Univariate linear and logistic regression analysis to predict readmissions and other postoperative outcomes.

	Readmission	Major Complications	Catheter Days	Hospital Stay	Symptomatic Lymphoceles	Positive Surgical Margins	Console Time	Transfusion
Prostate volume	0.447	0.390	0.953	0.778	0.654	0.684	0.005	0.212

## Data Availability

The data presented in this study are available on request from the corresponding author. The data are not publicly available due to the national data privacy regulations.
